# Supplementary material for: Humans and LLMs rate deliberation as superior to intuition on complex reasoning tasks
Source: Commun Psychol. 2025 Sep 29;3:141. doi: 10.1038/s44271-025-00320-8 (PMC12480527; doi:10.1038/s44271-025-00320-8)
Supplement: Supplementary file 2 — Supplementary Information [file 44271_2025_320_MOESM2_ESM.pdf]

Supplementary information

Contents

A. Demographic information about participants 2

B. Supplementary Methods: General material 3

Rating instructions 4

Ranking instructions 5

Debriefing 6

C. Supplementary Methods: LLM studies (8 and 9) 7

D. Supplementary Methods: Time-pressure studies (10 and 11) 8

Rating instructions 8

Ranking instructions 9

Trial exclusions 10

Rating 10

Ranking 10

E. Supplementary Methods: Two-response studies (12 and 13) 11

Material and procedure 11

Rating instructions 12

Ranking instructions 14

Trial exclusions 14

F. Correlations between scales 15

G. Complete ratings for studies 1-7 17

H. Between-subject analyses 19

I. Ranking profiles 21

J. Betting results 24

K. ChatGPT studies (8-9) 25

L. Ratings for deliberation restriction studies (10-13) 28

Supplementary references 31

A. Demographic information about participants

All tables in this section include information about participants who were later excluded from analyses.

Supplementary Table 1. Education.

Study	None	High school	Bachelor	Masters	PhD
1	1.7% (4)	35.7% (85)	42.0% (100)	17.2% (41)	3.4% (8)
2	2.1% (5)	32.8% (79)	39.4% (95)	22.8% (55)	2.9% (7)
3	1.2% (3)	42.1% (101)	35.8% (86)	18.3% (44)	2.5% (6)
4	0.0% (0)	32.9% (79)	50.4% (121)	13.8% (33)	2.9% (7)
5	0.8% (2)	31.5% (76)	51.0% (123)	13.7% (33)	2.9% (7)
6	1.7% (4)	17.5% (42)	28.7% (69)	49.6% (119)	2.5% (6)
7	0.0% (0)	9.8% (18)	48.9% (90)	36.4% (67)	4.9% (9)
10	1.2% (6)	38.0% (183)	46.4% (223)	12.3% (59)	2.1% (10)
11	0.4% (2)	31.7% (152)	50.1% (240)	15.2% (73)	2.5% (12)
12	0.4% (1)	28.3% (68)	53.3% (128)	15.4% (37)	2.5% (6)
13	1.2% (3)	29.2% (70)	49.2% (118)	17.1% (41)	3.3% (8)

Notes. Study labels: 1 = original, 2 = replication, 3 = accuracy, 4 = implicature, 5 = 1-scale, 6 = French, 7 = Indian, 10 = timing, 11 = timing-1-scale, 12 = two-response, 13 = two-response-hard. Education information was provided by participants by answering the following prompt: “What is the highest level of education you have completed? Less than High School / High School / GED /Bachelor Degree / Masters Degree / Doctoral Degree”. Overall, 2 participants didn’t disclose their education information.

Supplementary Table 2. Sample size per condition for time-pressure studies.

Study	Condition	n
10	Deadline	242
	Forced	240
11	Deadline	237
	Forced	242

Note. Study labels: 10 = timing, 11 = timing-1-scale.

B. Supplementary Methods: General material

Supplementary Table 3. Vignette texts.

Study	Reasoning mode	Accuracy	Description
All	Intuition	High	Person A follows their intuition when reasoning about a problem.<br>They do not spend much time or effort to arrive at a conclusion.<br>The accuracy of their answers is very high.
	Deliberation	High	Person B reflects deeply when reasoning about a problem.<br>They spend a lot of time and effort to arrive at a conclusion.<br>The accuracy of their answers is very high.
	Intuition	Low	Person C follows their intuition when reasoning about a problem.<br>They do not spend much time or effort to arrive at a conclusion.<br>The accuracy of their answers is very low.
	Deliberation	Low	Person D reflects deeply when reasoning about a problem.<br>They spend a lot of time and effort to arrive at a conclusion.<br>The accuracy of their answers is very low.
	Intuition	Unspecified	Person E follows their intuition when reasoning about a problem.<br>They do not spend much time or effort to arrive at a conclusion.
	Deliberation	Unspecified	Person F reflects deeply when reasoning about a problem.<br>They spend a lot of time and effort to arrive at a conclusion.
	Control		Person O.<br>There is no information about Person O's profile.
	Practice1		Person Z likes puzzles.<br>They also spend time solving crosswords.
10 – 13	Practice2		Person X is a fan of Sherlock Holmes.<br>They read a lot of detective novels, often finding out the identity of th[illegible]<br>perpetrator early.
	Practice3		Person Y has a background in mathematics.<br>They design encryption algorithms for a living.
3	Intuition	High	Person A follows their intuition when reasoning about a problem.<br>They do not spend much time or effort to arrive at a conclusion.<br>The accuracy of their answers is very high (95%).
	Deliberation	High	Person B reflects deeply when reasoning about a problem.<br>They spend a lot of time and effort to arrive at a conclusion.<br>The accuracy of their answers is very high (95%).
	Intuition	Low	Person C follows their intuition when reasoning about a problem.<br>They do not spend much time or effort to arrive at a conclusion.<br>The accuracy of their answers is very low (5%).
	Deliberation	Low	Person D reflects deeply when reasoning about a problem.<br>They spend a lot of time and effort to arrive at a conclusion.<br>The accuracy of their answers is very low (5%).
4	Intuition	High	Person A follows their intuition when reasoning about a problem.<br>They do not need to spend much time or effort to arrive at a conclusio[illegible]<br>The accuracy of their answers is very high.

Notes. The letters for each Person were randomized for each participant. All studies used practice item 1 while practice items 2 and 3 were only used in studies 10 – 13.

Rating instructions

After indicating their Prolific ID, participants were introduced to the rating task with the following instructions:

“Please read these instructions carefully.

We will give you information about the reasoning style of different individuals. We want to know how you evaluate their reasoning skills. Each time you will need to answer the following questions:”

A screenshot of the rating scales was displayed on the same page:

Supplementary Figure 1. Screenshot of the rating scales for the instructions.

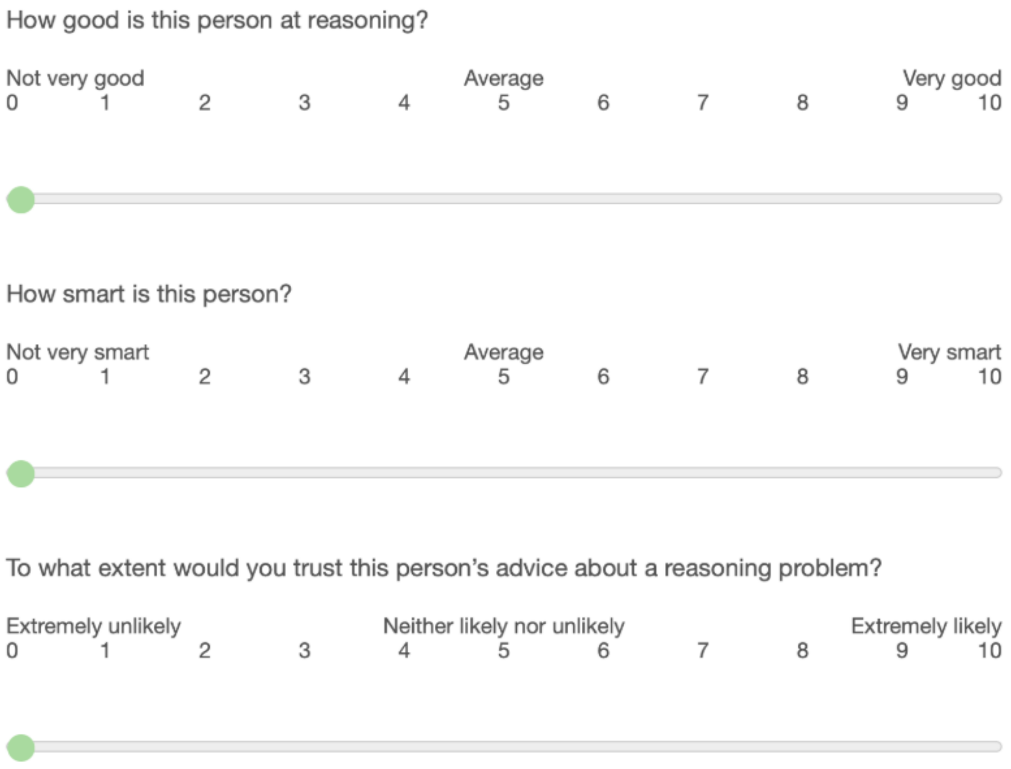

Note. For Studies 5 and 11 – 13, only one scale (How good is this person at reasoning?) was presented.

Instructions continued below the screenshot:

“You will need to click on the rating of your choice for each question and when you’re finished you can click on the next button.

We will show you an example next.”

This was followed with practice item 1 in all studies. For studies 10-13, two additional items were used to familiarize participants with the specific procedure involved (see the last two sections for details). After completing practice, participants proceeded to the main experiment.

Ranking instructions

The ranking task was introduced by the following instructions:

"*From the descriptions below, please now rank the people from the most intelligent (number 1) to the least intelligent (number 4).*

You can only place one person at each rank (i.e., no ties)."

The four vignettes were then displayed in a random order below the instructions along with buttons to indicate the ranking as pictured below:

Supplementary Figure 2. Screenshot of the ranking stage.

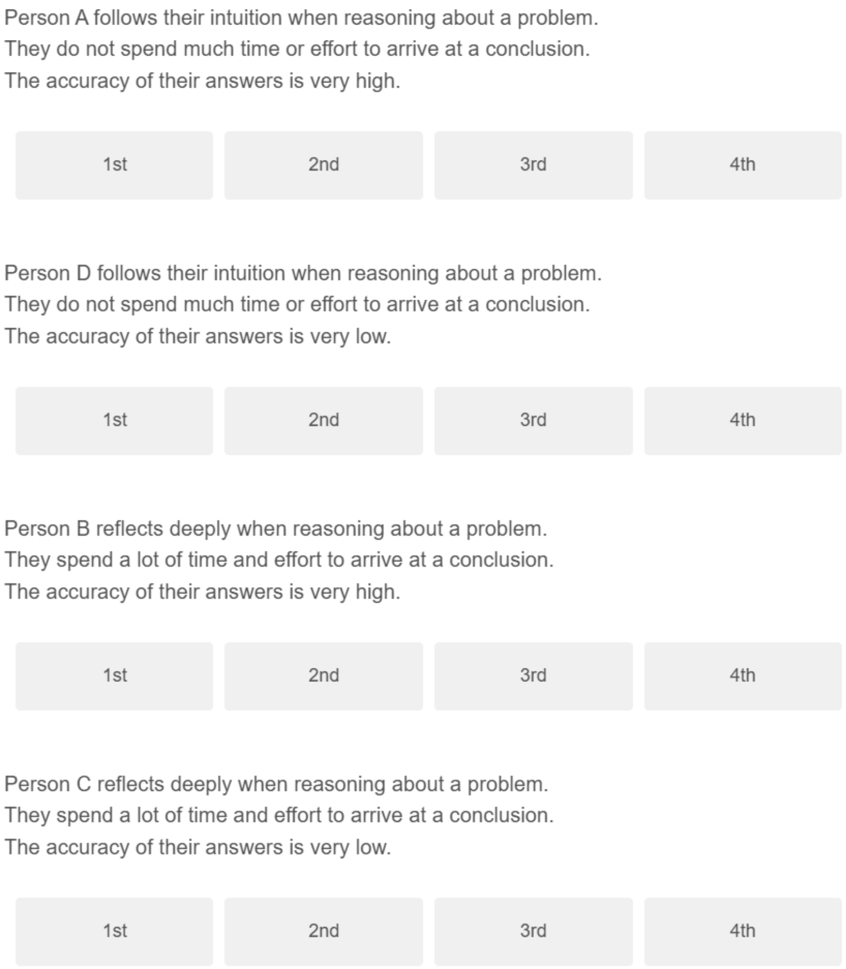

Note. The letter for each profile matched the one from the rating stage.

Additional instructions were displayed for Studies 10 – 13 (see the last two sections for details).

Debriefing

The following information was displayed at the end of each study:

"*The purpose of this experiment was to look at how a person's reasoning style influences our perception of them. For example, people may prefer others to make intuitive or deliberative decisions. We are interested in finding out which factors affect these preferences.*"

C. Supplementary Methods: LLM studies (8 and 9)

We used the *openai* package (Rudnytskyi, 2023) in R to query the Chat Completions API provided by OpenAI.

For each query, we used the following system role: "*You are a test subject in a psychology experiment. Your subject number is* ", which was completed by a number between 1 and 240.

For the message content, we used the following introduction:

"*I will give you information about the reasoning profile of 7 different individuals. I want you to rate these individuals on 3 separate scales based on their profile: 1) On a scale from 0 (not very good) to 10 (very good), how good is this person at reasoning? 2) On a scale from 0 (not very smart) to 10 (very smart), how smart is this person? 3) On a scale from 0 (extremely unlikely) to 10 (extremely likely), to what extent would you trust this person's advice about a reasoning problem?*
I need the results formated as in a tsv file with 7 rows (one for each profile) with the following headers:
-subject_number,
-profile,
-presentation_order,
-reasoning_scale,
-smart_scale,
-trust_scale.
Send it as a JSON object".

This introduction was followed by the 7 profiles presented in a pseudo-randomized order mimicking Study 1 conditions.

Finally, we added a formatting request to minimize inconsistencies in replies:

"*Please send me the formatted tsv for the 7 profiles as explained*".

We used the same procedure for both Studies, with different randomized orders of presentation. Data collection was completed on 25 March 2024 using gpt-3.5-turbo-0125 for Study 12 and on 25 June 2024 using gpt-4-turbo-2024-04-09 for Study 13.

Due to the randomness in responses (as we set the temperature to 1), replies didn't always have a rating available for each profile so some ratings were missing in the final dataset. Also note that our focus in these studies was on the ratings so we didn't try to reproduce RT or ranking data.

D. Supplementary Methods: Time-pressure studies (10 and 11)

The deadlines for Studies 10 and 11 were based on the first quartile of RT from Studies 1 (with 3 scales) and 5 (with 1 scale).

Supplementary Table 4. RT information for Studies 1, 5, 10 and 11

Study	*M*	*SD*	min	25%	50%	75%	max
1	20.6	23.2	3.5	12.0	15.8	21.9	421.1
5	12.1	16.3	1.4	6.3	8.9	13.0	385.9
10	9.9	1.8	3.4	8.6	10.1	11.7	12.2
11	5.2	1.0	1.6	4.4	5.5	6.0	6.2

Notes. Study labels: 1 = original, 5 = 1-scale, 10 = timing, 11 = timing-1-scale. Due to technical issues, the maximum value for RT reached beyond the deadline in Studies 10 and 11.

Rating instructions

After completing the regular practice (i.e., like in Study 1), new instructions were added to introduce the relevant timing manipulation.

Deadline condition:

“Please read these instructions carefully.

We are interested in your initial, intuitive response. We want you to indicate the very first evaluation that comes to mind.

You don't need to think about it. Just give the first rating that intuitively come to mind as quickly as possible for each scale.

To assure this, a time limit was set, which is going to be 12 seconds. When there are 3 seconds left, the background colour will change to let you know that the deadline is approaching.

Please make sure to answer before the deadline passes.

We will show you 2 examples to familiarize you with this procedure.”

Forced deliberation condition:

“Please read these instructions carefully.

We want to know how you evaluate profiles after you've carefully thought about them.

You will have as much time as you need.

To make sure that you are actually engaged in deliberation while you evaluate the profiles, you will be able to click on the rating of your choice on each scale but you will NOT be able to click on Next for the first 20 seconds.

Once the 20 seconds have passed and you have indicated your ratings on the different scales, the background colour will change to let you know that you can click on Next to validate your response if you are ready (but feel free to take more time if you need to).

We will show you 2 examples to familiarize you with this procedure."

Those instructions were similar in both studies except for the time limit in the deadline condition (i.e., 12 seconds and 6 seconds, respectively) and were followed by two practice items (i.e., Person X and Y from Table B1). Participants then proceeded to the main experiment.

Ranking instructions

Before proceeding to the ranking question, new instructions were presented to highlight the relevant time manipulations.

Deadline condition:

"*Please read these instructions carefully.*

For the next question, you will have to rank the profiles you have just seen.

You will have to rank people from the most intelligent (number 1) to the least intelligent (number 4).

Be careful: you can only place one Person at each rank (i.e., no ties).

As before, we are interested in your initial, intuitive response. We want you to indicate the very first ranking that comes to mind.

You don't need to think about it. Just give the first ranking that intuitively comes to mind as quickly as possible.

To assure this, a time limit was set, which is going to be 25 seconds. When there are 5 seconds left, the background colour will change to let you know that the deadline is approaching.

Please make sure to answer before the deadline passes."

Forced deliberation condition:

"*Please read these instructions carefully.*

For the next question, you will have to rank the profiles you have just seen.

You will have to rank people from the most intelligent (number 1) to the least intelligent (number 4).

Be careful: you can only place one Person at each rank (i.e., no ties).

As before, you will have as much time as you need. To make sure that you are actually engaged in deliberation while you rank the profiles, you will be able to click on the ranking of your choice but you will NOT be able to click on Next for the first 50 seconds.

Once the 50 seconds have passed and you have ranked the profiles, the background colour will change to let you know that you can click on Next to validate your response if you are ready (but feel free to take more time if you need to).”

Those instructions were similar in both studies.

Trial exclusions

Rating

For Study 10 we considered as missing trials where at least 2 values from the rating scales were not recorded (i.e., only 1 scale had a recorded a value or no scales at all). According to our preregistration, we removed 1 subject who had more than 2 trials missing. For the 481 remaining participants, we discarded 45 trials which were considered missing and replaced 81 single missing values with the sample mean for the relevant condition x item combination.

For Study 11 we considered as missing trials where no value was recorded. According to our preregistration, we removed 2 subjects who had more than 2 trials missing. For the 477 remaining participants, we replaced 88 single missing values with the sample mean for the relevant condition x item combination.

Ranking

Due to the timing conditions, we didn't force participants to complete the ranking task or prevent them from assigning tied ranks. As a result, 9 participants didn't complete the ranking and 25 participants had ties in their rankings in study 10. Similarly, 31 participants didn't complete the ranking and 23 participants had ties in their rankings in study 11. Those rankings were discarded from our analyses.

E. Supplementary Methods: Two-response studies (12 and 13)

Material and procedure

In the two-response paradigm (Bago & De Neys, 2017; Thompson et al., 2011), participants give an initial response immediately followed by a final response in a given trial. To minimize the involvement of deliberation in the initial response, the initial response is given under time pressure and cognitive load, whereas the final response is given under no constraint.

Figure E1 illustrates the patterns that participants had to keep in memory while initially evaluating a profile under a deadline (6 seconds and 5.5 seconds, respectively) in Studies 12 and 13. After giving their initial rating, participants had to recognize the memorized pattern among a set of 4 (the target pattern and 3 distractor patterns) chosen as follows:

- In Study 12, the first distractor shared 3 crosses with the target pattern, while the second distractor shared no crosses with the target pattern and the third distractor shared 3 crosses with the third distractor.
- In Study 13, the first distractor shared 3 crosses with the target pattern, while the second distractor shared 2 crosses with the target pattern and the third distractor shared 3 crosses with the third distractor.

Finally, participants could give their final response under no constraint. To visually distinguish the two response stages, the rating question ("*How good is this person at reasoning?*") was colored green or blue with a small text addition ("first hunch" or "think it through", respectively). Figure E2 illustrates a complete trial in Study 12.

Supplementary Figure 3. Examples of matrices for the two-response studies

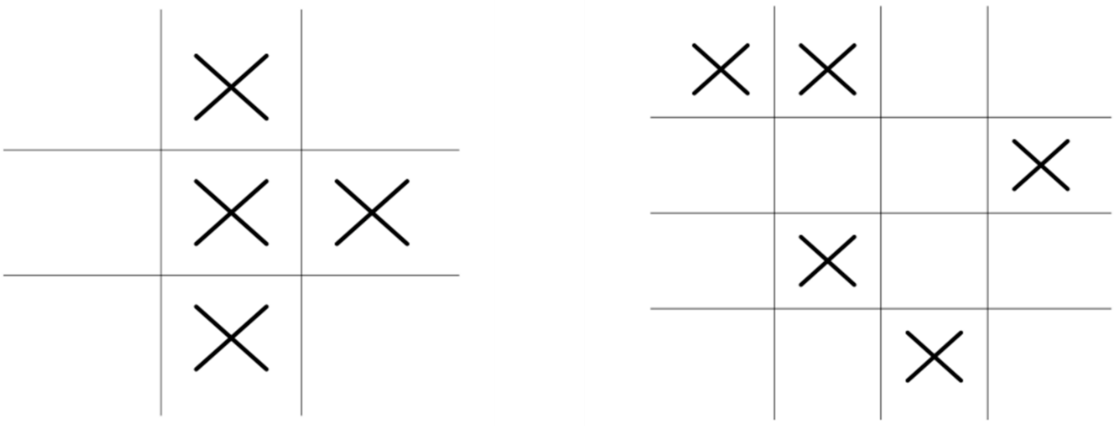

Note. Left: 4-cross pattern for Study 12. Right: 5-cross pattern for Study 13.

Supplementary Figure 4. Timeline for the two-response paradigm in Studies 12 and 13

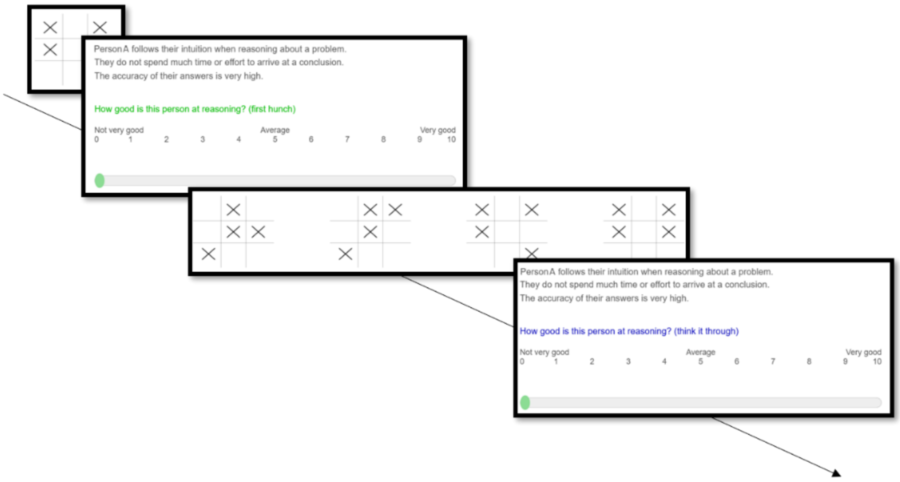

Notes. The pattern was presented for 2 seconds. Then, the vignette was presented with the rating scale until the deadline passed or the participant had selected their rating, whichever was the earliest. Participants then had to identify the previous pattern among a set of 4. Finally, the vignette was presented again with the rating scale under no constraint.

Rating instructions

After completing the regular practice (i.e., like in Study 1), new instructions were added to introduce the two-response paradigm:

"*Please read these instructions carefully.*

We are interested in your initial, first hunch as well as your evaluation after careful deliberation.

We first want you to indicate the very first evaluation that comes to mind.
You don't need to think about it. Just give the first rating that comes to mind as quickly as possible.
To assure this, a time limit was set, which is going to be 6 seconds. When there are 1.5 seconds left, the background colour will change to let you know that the deadline is approaching.

Please make sure to answer (i.e., click on your rating and click on Next) before the deadline passes.

After that, you will be able to take your time to think it through and evaluate the same profile again.

We will show you 2 examples next to familiarize you with the deadline first."

Those instructions were similar in both studies except for the time limit (i.e., 6 seconds and 5.5 seconds, respectively) and were followed by two practice items (i.e., Person X and Y from Table B1) under the respective deadline.

The load task was then introduced as follows:

"*Please read these instructions carefully.*

To make sure that your initial evaluation is the first that comes to your mind, we'll add a memorization task.

Before each profile, you'll first be presented with a pattern that you'll have to memorize.

After you have given your initial evaluation within the time limit, you'll have to identify this pattern among a set of 4.

Please focus on the memorization task.

We will first show you 2 examples of the memorization task."

Participants then had to rate the same two practice items while under the load task. Final instructions were then provided before rating the previous two practice items in the two-response paradigm:

"*Please read these instructions carefully.*

We will now show you 2 examples of the complete procedure.

Remember: you'll first have to give your initial evaluation, then identify the pattern. After that, you will be able to take your time to evaluate the same profile again."

Before starting the main experiment, participants were reminded of the paradigm one last time:

"*Remember: you'll first have to give your initial evaluation, then identify the pattern.*

We know it's not always easy to memorize the pattern and evaluate the profile at the same time.

Focus on the memory task and do your best for the initial evaluation.

After that, you will be able to take all the time you want to carefully evaluate the profile again."

Note that green and blue fonts were used in the relevant places to differentiate the two response stages in this set of instructions.

Ranking instructions

The ranking procedure was similar to Study 1, so the two-response paradigm didn't apply. Participants were therefore made aware of that with the following introduction before proceeding to the ranking question:

"Please read these instructions carefully.

For the next question, you will have to rank the profiles you have just seen.

You will have to rank people from the most intelligent (number 1) to the least intelligent (number 4).

You can take all the time you want for this.

Once you have ranked the profiles, you can click on Next to validate your response."

Trial exclusions

For both studies we considered as missing trials where no value was recorded for the initial response.

In Study 12, 110 trials out of 1680 (6.5%) were considered missing. We discarded data from 3 participants who had more than 2 trials missing.

Participants failed to complete the load task on 200 trials out of 1680 (11.9%). In line with our preregistration, we also discarded data from 2 participants who failed the load task on more than half their trials because we couldn't be sure that they had not already deliberated to produce their initial responses.

In Study 13, 121 trials out of 1680 (7.2%) were considered missing. We discarded data from 5 subjects who had more than 2 trials missing.

Participants failed to complete the load task on 491 trials out of 1680 (29.2%). In line with our preregistration, we also discarded data from 38 participants who failed the load task on more than half their trials because we couldn't be sure that they had not already deliberated to produce their initial responses.

We thus had 235 and 197 participants remaining, respectively. Single missing values were replaced with sample means.

F. Correlations between scales

Supplementary Figure 5. Rating scores on separate scales and across scales for Study 1.

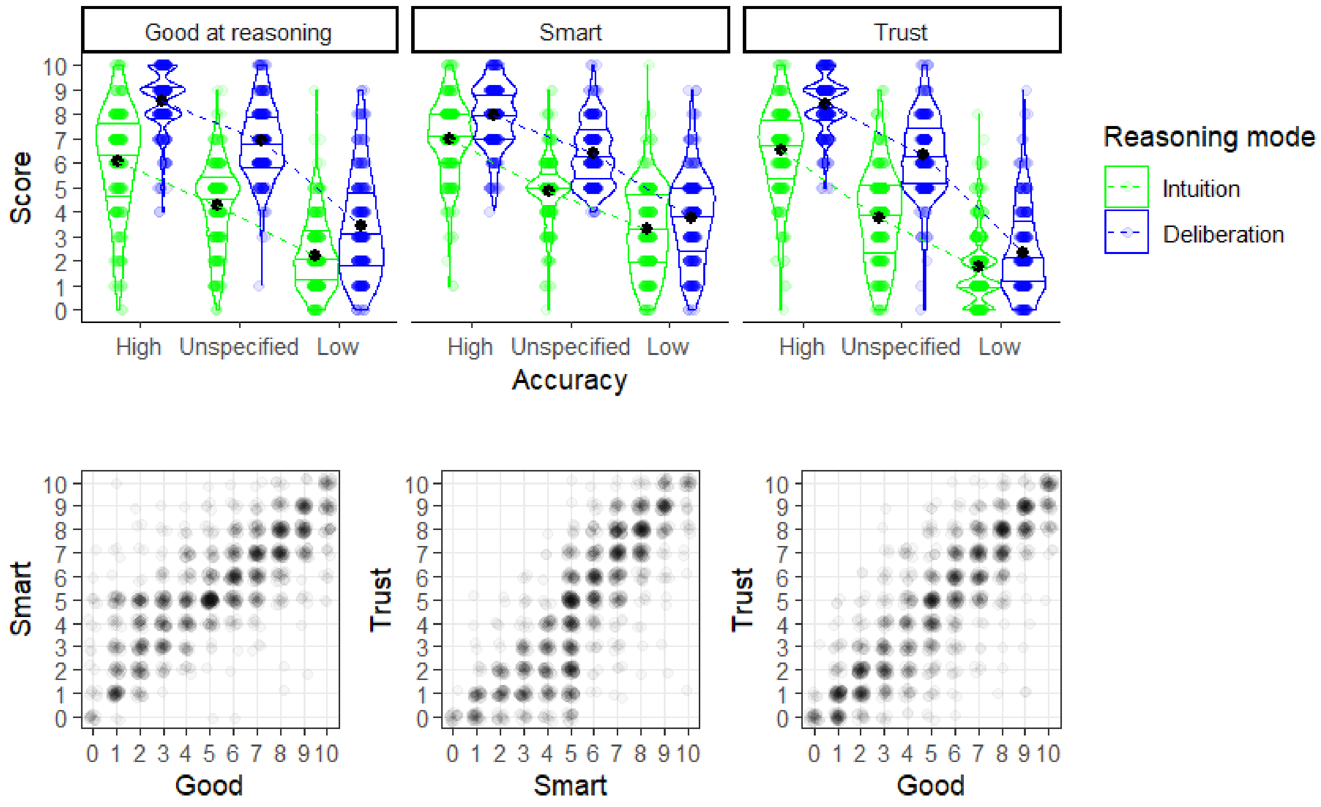

Notes. Top panel: violin plots indicate individual ratings for each profile, separated by scale. Black dots indicate the average scores. Bottom panel: scatterplots indicate individual ratings on each of 2 scales. *n* = 239 participants.

Supplementary Table 5. Correlations between rating scales for all studies.

		r		
Study	*df*	Good:Smart	Smart:Trust	Good:Trust
1	1432	.79 [.77, .81]	.83 [.82, .85]	.87 [.86, .88]
2	1444	.80 [.78, .82]	.84 [.82, .85]	.87 [.86, .89]
3	1438	.84 [.82, .85]	.87 [.86, .88]	.91 [.90, .92]
4	1438	.78 [.76, .80]	.85 [.84, .87]	.88 [.87, .89]
6	1438	.79 [.77, .81]	.80 [.78; .82]	.87 [.86, .89]
7	1102	.86 [.85, .88]	.86 [.84, .88]	.86 [.85, .88]
10:restricted	1421	.85 [.83, .86]	.84 [.82, .85]	.86 [.85, .87]
10:unrestricted	1426	.82 [.81, .84]	.85 [.83, .86]	.88 [.87, .90]

Notes. 1 = original (*n* = 239), 2 = replication (*n* = 241), 3 = accuracy (*n* = 240), 4 = implicature (*n* = 240), 5 = 1-scale (*n* = 241), 6 = French (*n* = 240), 7 = Indian (*n* = 184), 10 = timing (*n* = 241 and 240 with and without restriction, respectively). Some ratings were missing in the timing study so profiles where at least one rating was missing were not considered for correlations. All correlations were significant at $p < .001$. Square brackets indicate 95% CI.

G. Complete ratings for studies 1-7

Supplementary Figure 6. Combined rating scores for all items of Studies 1 – 7.

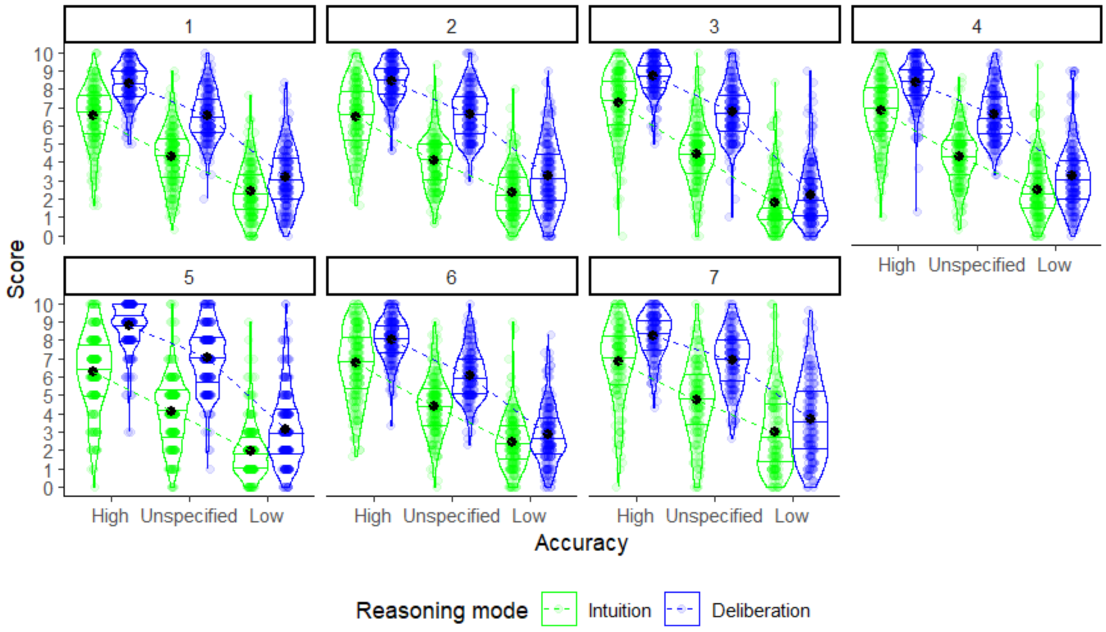

Notes. Study labels: 1 = original (n = 239), 2 = replication (n = 241), 3 = accuracy (n = 240), 4 = implicature (n = 240), 5 = 1-scale (n = 241), 6 = French (n = 240), 7 = Indian (n = 184). Black dots indicate the average scores. Horizontal lines represent the 25th, 50th and 75th percentiles).

Supplementary Table 6. Contrasts for within-subject ANOVAs for Studies 1-7. We looked at the contrast between Deliberation and Intuition at every Accuracy level. Deliberation was consistently rated higher than intuition.

Study	Accuracy	estimate	*SE*	*df*	*t*	*p*	*d*
1	High	1.75 [1.52, 1.97]	0.11	238	15.35	< .001	1.22 [1.03, 1.42]
	Unspecified	2.26 [2.03, 2.49]	0.12	238	19.30	< .001	1.58 [1.34, 1.80]
	Low	0.75 [0.56, 0.94]	0.10	238	7.69	< .001	0.53 [0.38, 0.67]
2	High	1.96 [1.71, 2.21]	0.13	240	15.37	< .001	1.33 [1.12, 1.53]
	Unspecified	2.51 [2.26, 2.75]	0.12	240	20.42	< .001	1.69 [1.47, 1.92]
	Low	0.89 [0.67, 1.11]	0.11	240	7.89	< .001	0.60 [0.44, 0.76]
3	High	1.49 [1.26, 1.71]	0.12	239	12.89	< .001	0.98 [0.81, 1.16]
	Unspecified	2.29 [2.02, 2.55]	0.13	239	17.00	< .001	1.51 [1.29, 1.74]
	Low	0.40 [0.23, 0.57]	0.08	239	4.73	< .001	0.27 [0.15, 0.38]
4	High	1.56 [1.32, 1.80]	0.12	239	12.79	< .001	1.03 [0.85, 1.22]
	Unspecified	2.33 [2.09, 2.58]	0.12	239	18.97	< .001	1.55 [1.34, 1.76]
	Low	0.75 [0.55, 0.96]	0.10	239	7.36	< .001	0.50 [0.36, 0.64]
5	High	2.52 [2.22, 2.82]	0.15	240	16.52	< .001	1.41 [1.20, 1.62]
	Unspecified	2.96 [2.61, 3.31]	0.18	240	16.65	< .001	1.66 [1.41, 1.91]
	Low	1.22 [0.96, 1.48]	0.13	240	9.25	< .001	0.68 [0.52, 0.84]
6	High	1.31 [1.05, 1.56]	0.13	239	10.08	< .001	0.87 [0.68, 1.06]
	Unspecified	1.64 [1.39, 1.89]	0.13	239	12.91	< .001	1.09 [0.90, 1.29]
	Low	0.39 [0.21, 0.57]	0.09	239	4.30	< .001	0.26 [0.14, 0.38]
7	High	1.43 [1.10, 1.76]	0.17	183	8.48	< .001	0.75 [0.56, 0.95]
	Unspecified	2.16 [1.79, 2.53]	0.19	183	11.49	< .001	1.14 [0.91, 1.36]
	Low	0.71 [0.38, 1.05]	0.17	183	4.19	< .001	0.38 [0.20, 0.56]

Notes. Study labels: 1 = original (*n* = 239), 2 = replication (*n* = 241), 3 = accuracy (*n* = 240), 4 = implicature (*n* = 240), 5 = 1-scale (*n* = 241), 6 = French (*n* = 240), 7 = Indian (*n* = 184). A Holm correction was applied separately for each study. Square brackets indicate 95% CI.

H. Between-subject analyses

Supplementary Figure 7. Combined rating scores for the first item of Study 1.

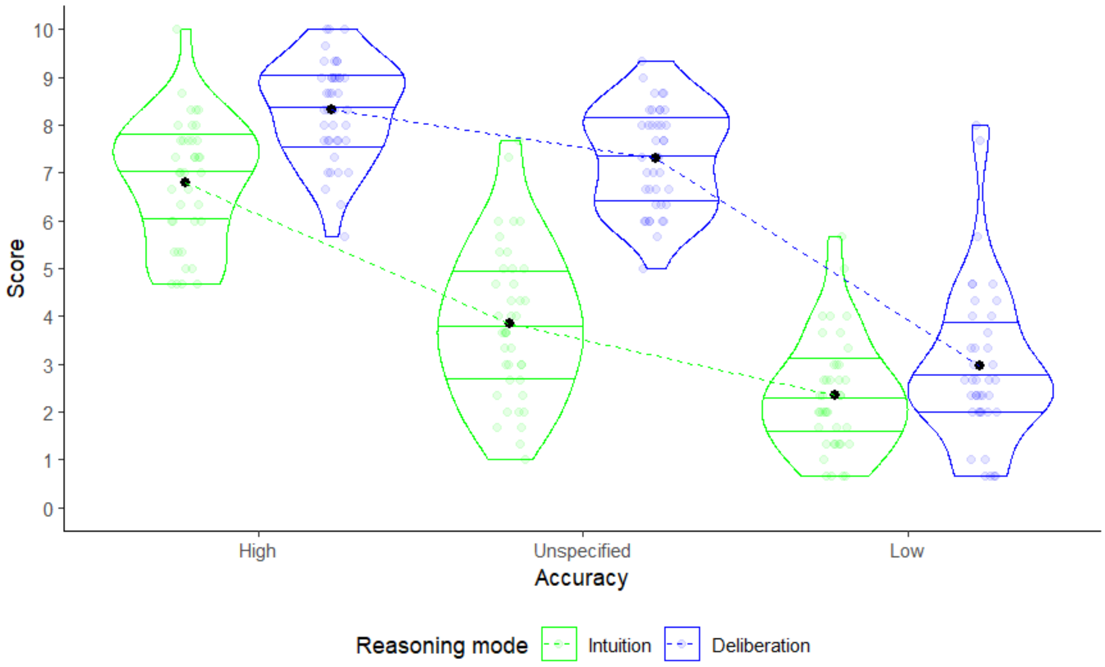

Notes. Black dots indicate the average scores. Horizontal lines represent the 25th, 50th and 75th percentiles). *n* = 239 participants.

Supplementary Table 7. Between-subject ANOVA for Study 1.

Effect	*df*	*F*	*p*	η_p^2
Mode	1, 233	117.68	<.001	.336
Accuracy	2, 233	273.07	<.001	.701
Mode:Accuracy	2, 233	23.76	<.001	.169

Supplementary Table 8. Contrasts for the between-subject ANOVA. We looked at the contrast between Deliberation and Intuition at every Accuracy level. Deliberation was consistently rated higher than intuition. *n* = 239 participants.

Accuracy	estimate	*SE*	*df*	*t*	*p*	*d*
High	1.51 [0.93, 2.1]	0.30	233	5.08	<.001	1.14 [0.69, 1.59]
Unspecified	3.47 [2.88, 4.05]	0.30	233	11.65	<.001	2.61 [2.10, 3.11]
Low	0.62 [0.03, 1.21]	0.30	233	2.08	.038	0.47 [0.02, 0.91]

Notes. A Holm correction was applied. Square brackets indicate 95% CI. *n* = 239 participants.

Supplementary Figure 8. Combined rating scores for the first item of Studies 1 – 7.

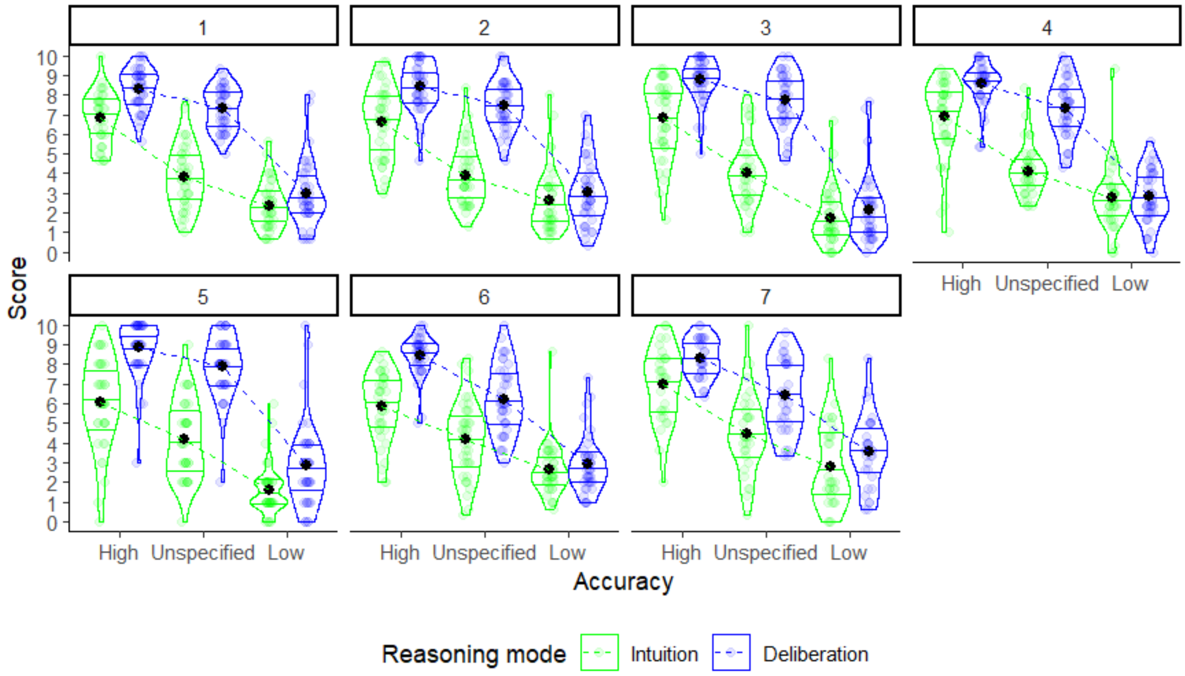

Notes. Study labels: 1 = original (*n* = 239), 2 = replication (*n* = 241), 3 = accuracy (*n* = 240), 4 = implicature (*n* = 240), 5 = 1-scale (*n* = 241), 6 = French (*n* = 240), 7 = Indian (*n* = 184). Black dots indicate the average scores. Horizontal lines represent the 25th, 50th and 75th percentiles).

I. Ranking profiles

Supplementary Figure 9. Ranking scores for Study 1.

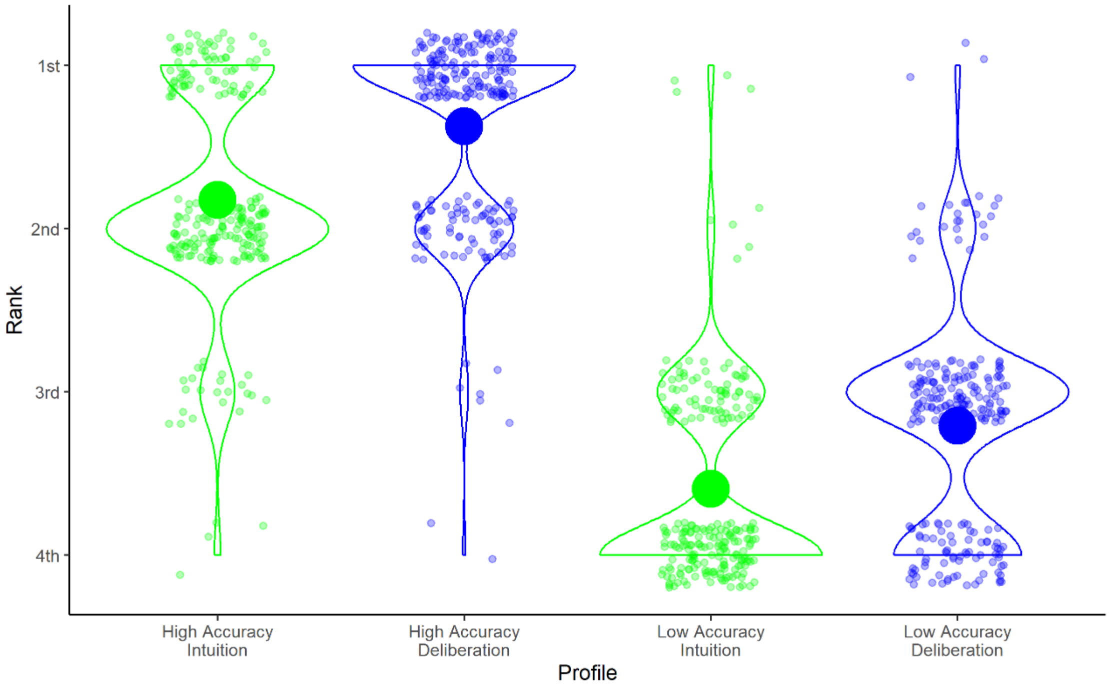

Notes. The bigger dots indicate the average rank for each profile. *n* = 239 participants.

Supplementary Table 9. Average ranks for Studies 1 – 7. On average, deliberation was consistently ranked higher than intuition, both for high accuracy and low accuracy, except for Study 6 (French sample).

	High accuracy		Low accuracy	
Study	Intuition	Deliberation	Intuition	Deliberation
1	1.82	1.37	3.59	3.21
2	1.84	1.37	3.58	3.21
3	1.75	1.44	3.52	3.29
4	1.76	1.45	3.52	3.27
5	1.96	1.37	3.61	3.06
6	1.88	1.92	3.15	3.06
7	1.92	1.46	3.56	3.06

Notes. Study labels: 1 = original (*n* = 239), 2 = replication (*n* = 241), 3 = accuracy (*n* = 240), 4 = implicature (*n* = 240), 5 = 1-scale (*n* = 241), 6 = French (*n* = 240), 7 = Indian (*n* = 184).

Supplementary Figure 10. Proportion of each ranking pattern for Studies 1 – 7.

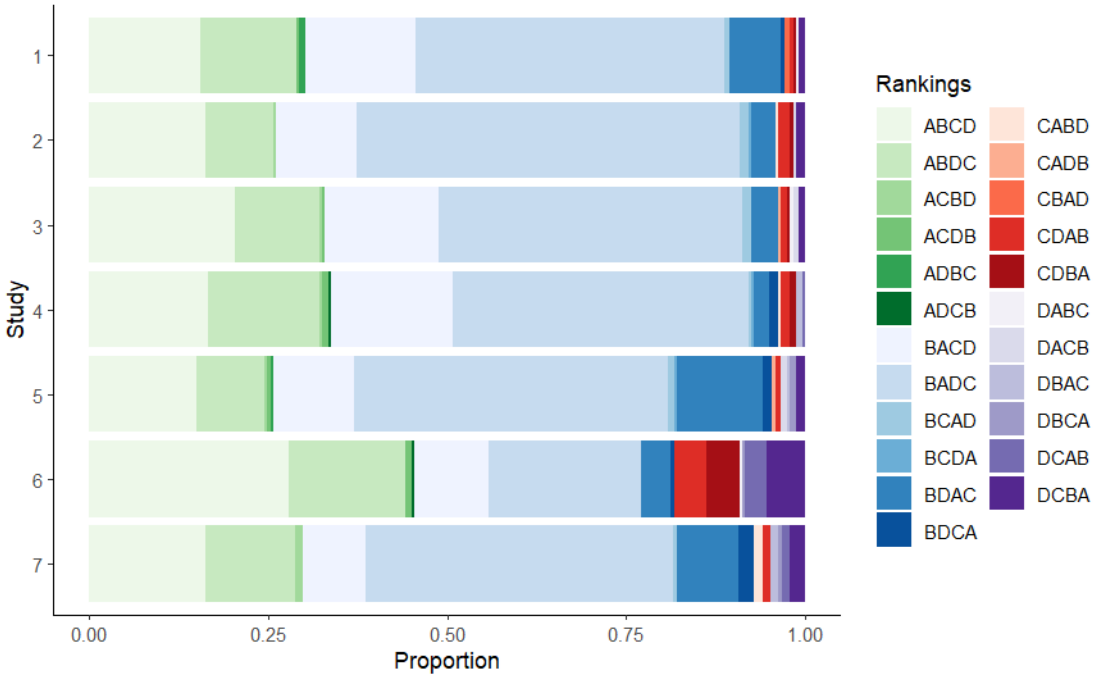

Notes. Study labels: 1 = original (*n* = 239), 2 = replication (*n* = 241), 3 = accuracy (*n* = 240), 4 = implicature (*n* = 240), 5 = 1-scale (*n* = 241), 6 = French (*n* = 240), 7 = Indian (*n* = 184). Ranking labels: A = High accuracy, intuition, B = high accuracy, deliberation, C = low accuracy, intuition, D = low accuracy, deliberation. Ranking patterns likely to represent (inattention) errors are also included for completeness. Most rankings favored high accuracy and deliberation (i.e., starting with B), followed by high accuracy and intuition (i.e., starting with A).

Supplementary Figure 11. Overall preference for deliberation or intuition for Studies 1 – 7.

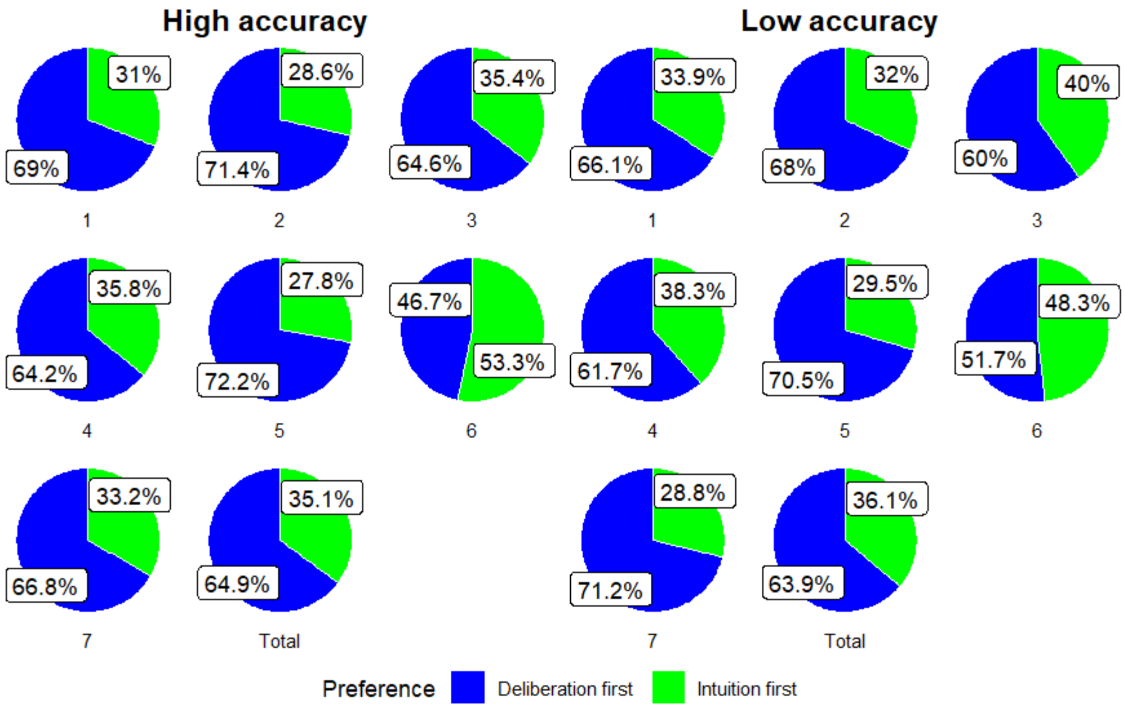

Note. Study labels: 1 = original (*n* = 239), 2 = replication (*n* = 241), 3 = accuracy (*n* = 240), 4 = implicature (*n* = 240), 5 = 1-scale (*n* = 241), 6 = French (*n* = 240), 7 = Indian (*n* = 184).

J. Betting results

Supplementary Table 10. Overall bets.

Bet	n	percent
A	52	21.6%
B	185	76.8%
C	2	0.8%
D	2	0.8%

Note. Ranking labels: A = High accuracy, intuition, B = high accuracy, deliberation, C = low accuracy, intuition, D = low accuracy, deliberation. n = 241 participants.

Supplementary Table 11. Bets according to the preference for deliberation or intuition with high accuracy.

	Bet			
Ranking preference	A	B	C	D
deliberation first	11.6% (20)	86.6% (149)	0.6% (1)	1.2% (2)
intuition first	46.4% (32)	52.2% (36)	1.4% (1)	0.0% (0)

Note. Ranking labels: A = High accuracy, intuition, B = high accuracy, deliberation, C = low accuracy, intuition, D = low accuracy, deliberation. n = 241 participants.

K. ChatGPT studies (8-9)

Supplementary Figure 12. Combined rating scores for all items in Studies 1 - 7, 8 and 9.

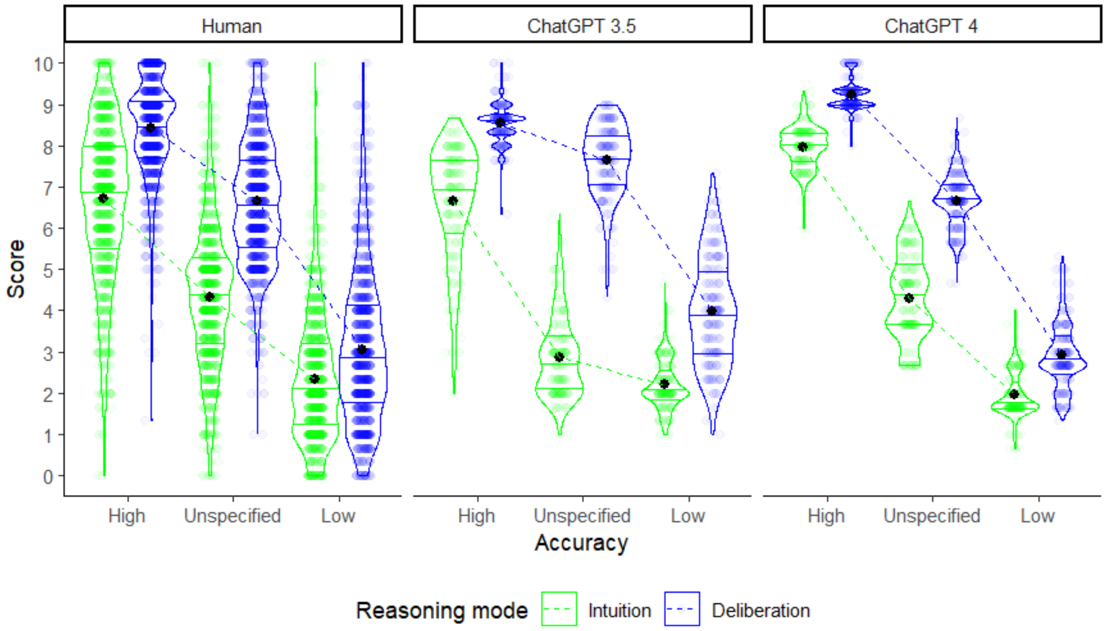

Notes. Black dots indicate the average scores. Horizontal lines represent the 25th, 50th and 75th percentiles). Ratings from Studies 1 – 7 were gathered into the Human label (*n* = 1625). For ChatGPT studies, *n* = 240 each.

Supplementary Table 12. Within-subject ANOVA (and sphericity) table for Studies 8 and 9.

		ANOVA					Sphericity		
Study	Effect	DFn	DFd	*F*	*p*	η_p^2s	W	*p*	$\hat{\varepsilon}$
8	Mode	1.00	224.00	2501.09	<.001	.918			
	Accuracy	2.00	448.00	2515.03	<.001	.918	1	.956	1
	Mode:Accuracy	1.86	417.22	394.99	<.001	.638	0.93	<.001	0.93
9	Mode	1.00	236.00	1726.12	<.001	.88			
	Accuracy	2.00	472.00	8714.43	<.001	.974	0.98	.089	0.98
	Mode:Accuracy	1.91	449.60	196.32	<.001	.454	0.95	.003	0.95

Notes. Study labels: 8 = ChatGPT 3.5, 9 = ChatGPT 4 (*n* = 240 each). The Greenhouse-Geisser correction is only applied to *df* when the sphericity assumption is violated.

Supplementary Table 13. Contrasts for within-subject ANOVAs for Studies 8 and 9. We looked at the contrast between Deliberation and Intuition at every Accuracy level. As with human participants, deliberation was consistently rated higher than intuition.

Study	Accuracy	estimate	*SE*	*df*	*t*	*p*	*d*
8	High	1.93 [1.74, 2.12]	0.10	224	19.81	<.001	1.92 [1.66, 2.18]
	Unspecified	4.79 [4.62, 4.96]	0.08	224	56.97	<.001	4.77 [4.29, 5.24]
	Low	1.81 [1.64, 1.98]	0.09	224	20.83	<.001	1.80 [1.56, 2.04]
9	High	1.27 [1.21, 1.34]	0.03	236	39.48	<.001	1.89 [1.69, 2.08]
	Unspecified	2.36 [2.21, 2.50]	0.07	236	32.62	<.001	3.49 [3.11, 3.87]
	Low	0.97 [0.86, 1.08]	0.06	236	17.02	<.001	1.44 [1.23, 1.65]

Notes. Study labels: 8 = ChatGPT 3.5, 9 = ChatGPT 4 (*n* = 240 each). A Holm correction was applied separately for each study. Square brackets indicate 95% CI.

Supplementary Table 14. Average combined rating (and SD) for all items of Studies 1 – 9.

	Intuition			Deliberation		
Study	High accuracy	Unspecified accuracy	Low accuracy	High accuracy	Unspecified accuracy	Low accuracy
1	6.53 (1.59)	4.29 (1.53)	2.41 (1.42)	8.28 (1.05)	6.55 (1.29)	3.16 (1.59)
2	6.5 (1.79)	4.11 (1.44)	2.34 (1.32)	8.46 (1.04)	6.62 (1.34)	3.23 (1.8)
3	7.22 (1.75)	4.46 (1.7)	1.83 (1.42)	8.7 (0.98)	6.75 (1.45)	2.23 (1.63)
4	6.83 (1.73)	4.27 (1.47)	2.49 (1.49)	8.39 (1.18)	6.6 (1.36)	3.24 (1.73)
5	6.29 (2.15)	4.1 (1.96)	1.99 (1.49)	8.81 (1.25)	7.07 (1.72)	3.2 (1.99)
6	6.76 (1.9)	4.43 (1.58)	2.46 (1.41)	8.07 (1.12)	6.08 (1.35)	2.85 (1.55)
7	6.85 (2.1)	4.74 (2.06)	2.98 (2.16)	8.28 (1.19)	6.89 (1.55)	3.69 (2.12)
8	6.66 (1.39)	2.86 (0.97)	2.21 (0.6)	8.55 (0.47)	7.65 (0.88)	3.99 (1.3)
9	7.96 (0.52)	4.3 (1.01)	1.95 (0.54)	9.23 (0.35)	6.66 (0.63)	2.92 (0.79)
Average for humans	6.7 (1.88)	4.33 (1.69)	2.33 (1.56)	8.43 (1.14)	6.64 (1.47)	3.07 (1.82)
Average for ChatGPT	7.31 (1.23)	3.59 (1.22)	2.08 (0.58)	8.89 (0.54)	7.14 (0.91)	3.46 (1.2)

Note. Study labels: 1 = original (*n* = 239), 2 = replication (*n* = 241), 3 = accuracy (*n* = 240), 4 = implicature (*n* = 240), 5 = 1-scale (*n* = 241), 6 = French (*n* = 240), 7 = Indian (*n* = 184), 8 = ChatGPT 3.5 (*n* = 240), 9 = ChatGPT 4 (*n* = 240).

Supplementary Table 15. Average combined rating (and SD) for the first item of Studies 1 – 9.

	Intuition			Deliberation		
Study	High accuracy	Unspecified accuracy	Low accuracy	High accuracy	Unspecified accuracy	Low accuracy
1	6.8 (1.3)	3.84 (1.6)	2.34 (1.18)	8.32 (1.04)	7.31 (1.06)	2.97 (1.68)
2	6.63 (1.78)	3.87 (1.53)	2.65 (1.62)	8.46 (1.2)	7.47 (1.25)	3.05 (1.64)
3	6.83 (1.94)	4.06 (1.74)	1.74 (1.38)	8.77 (1.1)	7.7 (1.39)	2.12 (1.7)
4	6.87 (1.99)	4.13 (1.26)	2.78 (1.6)	8.56 (1.09)	7.32 (1.42)	2.85 (1.33)
5	6.1 (2.28)	4.21 (1.98)	1.68 (1.29)	8.88 (1.44)	7.93 (1.62)	2.88 (2.17)
6	5.9 (1.72)	4.17 (1.9)	2.61 (1.31)	8.45 (1.04)	6.23 (1.73)	2.91 (1.45)
7	7.02 (1.89)	4.51 (2.04)	2.83 (2.09)	8.32 (1)	6.44 (1.85)	3.56 (1.81)
8	7.45 (0.58)	2.53 (0.56)	2.07 (0.48)	8.41 (0.45)	8.18 (0.44)	3.97 (1.4)
9	7.97 (0.48)	2.86 (0.38)	1.67 (0.32)	9 (0.13)	6.85 (0.41)	2.58 (0.74)
Average for humans	6.58 (1.89)	4.1 (1.72)	2.36 (1.54)	8.55 (1.15)	7.23 (1.58)	2.89 (1.72)
Average for ChatGPT	7.76 (0.58)	2.73 (0.48)	1.91 (0.47)	8.74 (0.43)	7.19 (0.72)	3.35 (1.35)

Note. Study labels: 1 = original (*n* = 239), 2 = replication (*n* = 241), 3 = accuracy (*n* = 240), 4 = implicature (*n* = 240), 5 = 1-scale (*n* = 241), 6 = French (*n* = 240), 7 = Indian (*n* = 184), 8 = ChatGPT 3.5 (*n* = 240), 9 = ChatGPT 4 (*n* = 240).

L. Ratings for deliberation restriction studies (10-13)

Supplementary Figure 13. Combined rating scores for all items of Studies 10 – 13.

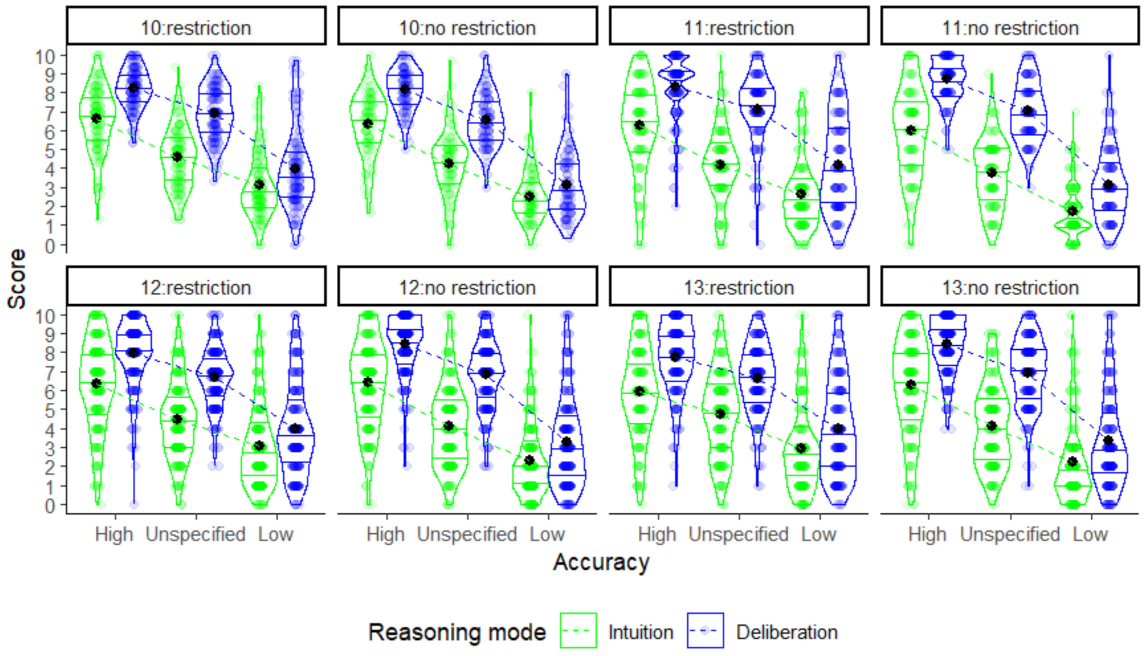

Notes. Study labels: 10 = timing (*n* = 241 and 240 with and without restriction, respectively), 11 = timing-1-scale (*n* = 236 and 241 with and without restriction, respectively), 12 = two-response (*n* = 235), 13 = two-response-hard (*n* = 197). Black dots indicate the average scores. Horizontal lines represent the 25th, 50th and 75th percentiles).

Supplementary Table 16. Contrasts for within-subject ANOVAs for Studies 10-13. We looked at the contrast between Deliberation and Intuition at every Accuracy level. Deliberation was consistently rated higher than intuition.

Study	Accuracy	Restriction	estimate	*SE*	*df*	*t*	*p*	BF_{10}	*d*
10	High	Restricted	1.53 [1.27, 1.79]	0.13	446	11.61	<.001	1.10×10^{21}	1.00 [0.82, 1.18]
	Unspecified	Restricted	2.31 [2.07, 2.56]	0.13	446	18.36	<.001	1.21×10^{40}	1.51 [1.32, 1.70]
	Low	Restricted	0.75 [0.55, 0.96]	0.10	446	7.18	<.001	1.7×10^{7}	0.49 [0.35, 0.63]
	High	Unrestricted	1.73 [1.47, 1.98]	0.13	446	13.41	<.001	6.89×10^{27}	1.13 [0.95, 1.31]
	Unspecified	Unrestricted	2.44 [2.20; 2.68]	0.12	446	19.78	<.001	1.22×10^{51}	1.59 [1.40, 1.78]
	Low	Unrestricted	0.57 [0.37, 0.77]	0.10	446	5.56	<.001	3.4×10^{6}	0.37 [0.24, 0.51]
11	High	Restricted	2.06 [1.74, 2.38]	0.16	475	12.69	<.001	5.47×10^{23}	1.10 [0.92, 1.29]
	Unspecified	Restricted	2.87 [2.54, 3.20]	0.17	475	17.04	<.001	5.70×10^{36}	1.54 [1.33, 1.74]
	Low	Restricted	1.30 [1.01, 1.59]	0.15	475	8.68	<.001	1.61×10^{10}	0.70 [0.53, 0.86]
	High	Unrestricted	2.54 [2.23, 2.86]	0.16	475	15.84	<.001	9.22×10^{37}	1.36 [1.17, 1.55]
	Unspecified	Unrestricted	3.20 [2.88, 3.53]	0.17	475	19.24	<.001	4.42×10^{50}	1.72 [1.51, 1.92]
	Low	Unrestricted	1.28 [0.99, 1.57]	0.15	475	8.65	<.001	1.33×10^{17}	0.69 [0.52, 0.85]
12	High	Restricted	1.62 [1.28, 1.96]	0.17	234	9.38	<.001	8.15×10^{14}	0.80 [0.62, 0.98]
	Unspecified	Restricted	2.24 [0.92, 2.56]	0.16	234	13.70	<.001	3.2×10^{28}	1.11 [0.92, 1.29]
	Low	Restricted	0.91 [0.56, 1.27]	0.18	234	5.04	<.001	$1.1\text{x}10^{4}$	0.45 [0.27, 0.63]
	High	Unrestricted	2.07 [1.72, 2.42]	0.18	234	11.54	<.001	3.48×10^{21}	1.02 [0.82, 1.22]
	Unspecified	Unrestricted	2.70 [2.32, 3.09]	0.20	234	13.83	<.001	8.61×10^{28}	1.33 [1.11, 1.56]
	Low	Unrestricted	0.97 [0.68, 1.27]	0.15	234	6.52	<.001	1.74×10^{7}	0.48 [0.33, 0.63]
13	High	Restricted	1.83 [1.44, 2.22]	0.20	196	9.28	<.001	1.61×10^{14}	0.85 [0.65, 1.05]
	Unspecified	Restricted	1.89 [1.51, 2.28]	0.20	196	9.59	<.001	1.21×10^{15}	0.88 [0.68, 1.08]

Study	Accuracy	Restriction	estimate	*SE*	*df*	*t*	*p*	*BF₁₀*	*d*
	Low	Restricted	1.06 [0.70, 1.43]	0.18	196	5.74	<.001	$2.68x10^{5}$	0.49 [0.32, 0.67]
	High	Unrestricted	2.16 [1.78, 2.54]	0.19	196	11.26	<.001	$7.60×10^{19}$	1.00 [0.80, 1.20]
	Unspecified	Unrestricted	2.80 [2.38, 3.22]	0.21	196	13.21	<.001	$4.94×10^{25}$	1.30 [1.06, 1.53]
	Low	Unrestricted	1.11 [0.76, 1.47]	0.18	196	6.21	<.001	2.78×10	0.52 [0.34, 0.69]

Notes. Study labels: 10 = timing (*n* = 241 and 240 with and without restriction, respectively), 11 = timing-1-scale (*n* = 236 and 241 with and without restriction, respectively), 12 = two-response (*n* = 235), 13 = two-response-hard (*n* = 197). Square brackets indicate 95% CI. The BF_{10} column indicates the Bayes factor obtained from Bayesian paired t-tests run in JASP [4] with default priors. Higher values indicate increasing evidence in favor of a deliberation preference at the given Accuracy x Restriction level.

Supplementary Table 17. Average combined rating (and SD) for all items of Studies 10 – 13.

		Intuition			Deliberation		
Study	Restriction	Sys1_High	Sys1_No	Sys1_Low	Sys2_High	Sys2_No	Sys2_Low
10	Restricted	6.52 (1.79)	4.55 (1.59)	3.07 (1.66)	8.09 (1.21)	6.81 (1.37)	3.89 (2.06)
	Unrestricted	6.47 (1.85)	4.02 (1.51)	2.36 (1.32)	8.25 (1.07)	6.53 (1.31)	2.96 (1.63)
11	Restricted	6.28 (2.21)	4.23 (1.99)	2.63 (1.92)	8.34 (1.51)	7.09 (1.85)	3.93 (2.42)
	Unrestricted	6.2 (2.24)	3.8 (1.74)	1.84 (1.37)	8.74 (1.13)	7.01 (1.59)	3.12 (2.03)
12	Restricted	6.38 (2.31)	4.47 (2)	3.08 (2.14)	8 (1.69)	6.71 (1.51)	4 (2.39)
	Unrestricted	6.4 (2.3)	4.14 (2.15)	2.3 (1.85)	8.47 (1.52)	6.85 (1.73)	3.27 (2.4)
13	Restricted	5.92 (2.39)	4.74 (2.22)	2.95 (2.23)	7.75 (1.81)	6.64 (1.82)	4.01 (2.52)
	Unrestricted	6.26 (2.52)	4.15 (2.15)	2.23 (2.22)	8.43 (1.44)	6.95 (1.82)	3.35 (2.47)

Note: Study labels: 10 = timing (*n* = 241 and 240 with and without restriction, respectively), 11 = timing-1-scale (*n* = 236 and 241 with and without restriction, respectively), 12 = two-response (*n* = 235), 13 = two-response-hard (*n* = 197).

Supplementary references

1. Rudnytskyi, I. openai: R Wrapper for OpenAI API. R package version 0.4.1 (2023). Available at: https://CRAN.R-project.org/package=openai.
2. Bago, B., & De Neys, W. Fast logic?: Examining the time course assumption of dual process theory. *Cognition*, **158**, 90-109. https://doi.org/10.1016/j.cognition.2016.10.014 (2017).
3. Thompson, V. A., Turner, J. A. P., & Pennycook, G. Intuition, reason, and metacognition. *Cognitive Psychology*, **63**, 107–140. https://doi.org/10.1016/j.cogpsych.2011.06.001 (2011).
4. JASP Team. *JASP (Version 0.19.3)* 2025. Available at: https://jasp-stats.org/.
